# Treatment of patients with type 2 diabetes with exenatide once weekly versus oral glucose-lowering medications or insulin glargine: achievement of glycemic and cardiovascular goals

**DOI:** 10.1186/1475-2840-12-48

**Published:** 2013-03-23

**Authors:** Alison R Meloni, Mary Beth DeYoung, Jenny Han, Jennie H Best, Michael Grimm

**Affiliations:** 1 Amylin Pharmaceuticals, LLC, 9360 Towne Centre Drive, San Diego, CA, 92121, USA

**Keywords:** Number needed to treat, Absolute benefit, Exenatide, Type 2 diabetes, Diabetes mellitus, ADA treatment guidelines, GLP-1

## Abstract

**Background:**

Diabetes is associated with a higher risk for adverse cardiovascular outcomes. To improve the health outcomes of patients with type 2 diabetes (T2DM), the American Diabetes Association (ADA) recommended target goals for the improvement of glycemic control and the reduction of cardiovascular risk factors associated with the disease. This retrospective analysis calculated the absolute benefit increase (ABI) of using exenatide once weekly (QW), a glucagon-like peptide-1 (GLP-1) receptor agonist, vs an oral glucose-lowering medication or insulin glargine to achieve ADA-recommended goals. The number needed to treat (NNT) to achieve these goals was also calculated and provides a useful clinical metric for comparing potential therapies from different drug classes.

**Methods:**

Patient data from three double-blind or open label, 26-week, randomized, controlled trials were retrospectively analyzed separately. ABI and NNT were calculated by comparing the percentage of patients treated with exenatide QW (N = 641) vs metformin (N = 246), sitagliptin (N = 329), pioglitazone (N = 328), or insulin glargine (N = 223), who achieved a single glycemic, weight, blood pressure, or lipid goal or a composite of these recommended goals, during the DURATION-2, -3, and -4 clinical trials.

**Results:**

Significant ABIs favoring exenatide QW over all four glucose-lowering medications were observed for at least one HbA1c glycemic goal. NNTs of 4 and 5 were calculated when exenatide QW was compared to sitagliptin for attaining HbA1c goals of <7.0% and ≤6.5%, respectively. Additionally, significantly more patients using exenatide QW compared to sitagliptin, pioglitazone, or insulin glargine attained the composite goal of HbA1c <7% or ≤6.5%, without weight gain or hypoglycemia. Exenatide QW was also favored over sitagliptin and insulin glargine for the achievement of the composite goals of HbA1c <7% (or ≤6.5%), systolic blood pressure <130 mm Hg, and low-density lipoprotein <2.59 mmol/L. For most goals, exenatide QW and metformin had similar effects in treatment naïve patients.

**Conclusions:**

This analysis assessed the between-therapy differences in achieving therapeutic goals with therapies commonly used for glycemic control in patients with T2DM. In clinical trials, exenatide QW assisted more patients in reaching the majority of ADA-recommended therapeutic goals than treatment with sitagliptin, pioglitazone, or insulin glargine.

**Trial registration:**

NCT00637273, NCT00641056, NCT00676338

## Background

Although the primary goal in treatment of type 2 diabetes mellitus (T2DM) is the reduction of hyperglycemia, significant benefits of glycemic control on cardiovascular disease (CVD) in patients with T2DM were observed during the UK Prospective Diabetes Study (UKPDS), which showed a 14% decrease in the risk of myocardial infarction and 12% decrease in risk of stroke for each 1% decrease in glycated hemoglobin A1c (HbA1c) [1]. While reduction of hyperglycemia is clearly beneficial, avoidance of hypoglycemia is also a critical concern, as severe hypoglycemia was associated with an increased risk of death during the ACCORD study [2].

Long-term management of patients with T2DM should target not only glycemic control but also cardiovascular (CV) risk factors such as blood pressure, body weight, and lipids. The Steno-2 study examined a multifactorial approach to diabetes treatment, targeting HbA1c, blood pressure, lipids, and lifestyle modifications in an intensive intervention to prevent CVD in patients with T2DM [3,4]. Compared to conventional therapy, intensive multifactorial intervention reduced the risk of CV and microvascular events by approximately 50%. Based on these and other well-controlled and uncontrolled clinical studies, (Steno-2 [4], ADVANCE [5], ACCORD [6], UKPDS [7,8], DCCT [9], The Kumamoto Study [10] and others [11]) the American Diabetes Association (ADA) developed guidelines providing treatment goals intended to offer health benefits for patients with T2DM who are able to achieve these goals (Table 1) [11].

**Table 1:** Therapeutic goals explored in the analysis

**Selected ADA goals**	**Composite of Selected ADA Goals**
HbA1c <7.0%	HbA1c <7.0%, no weight gain, and no hypoglycemia†
*HbA1c ≤6.5%	HbA1c ≤6.5%, no weight gain, and no hypoglycemia†
**FBG <6.99 mmol/L	HbA1c <7.0%, SBP <130 mm Hg, and LDL <2.59 mmol/L
No weight gain	HbA1c ≤6.5%, SBP <130 mm Hg, and LDL <2.59 mmol/L
Any weight loss	
No hypoglycemia†	
SBP <130 mm Hg	
LDL <2.59 mmol/L	

*Actual ADA goal is <6.5% if it can be achieved without significant hypoglycemia.

**Not an ADA goal (diabetes diagnosis).

†Hypoglycemia is defined as documented blood glucose <3 mmol/L (<54 mg/dL).

Glucose conversion factor, 0.05551; LDL conversion factor, 0.02586.

FBG, fasting blood glucose; LDL, low density lipoprotein cholesterol; SBP, systolic blood pressure.

The GLP-1 receptor agonist, exenatide, is a synthetic peptide that has been shown not only to reduce hyperglycemia in patients with T2DM but also to improve body weight, blood pressure, and lipid profiles [12-16]. Exenatide twice daily has been shown to not increase the risk of CV events in a pooled analysis of clinical trial data.[17] A database analysis using the real-world data has demonstrated that exenatide twice daily treatment was associated with a lower risk of CVD events and hospitalizations than treatment with other glucose-lowering therapies.[18] The most recently approved formulation of exenatide, exenatide once weekly (QW) for subcutaneous injection, provides a slow release of exenatide from poly-(d,l-lactide-co-glycolide) microspheres, allowing weekly dosing [19]. Head-to-head comparisons have been made between exenatide QW and other GLP-1 receptor agonist therapies such as exenatide twice daily and liraglutide [20-22], as well as non-GLP-1 receptor agonist therapies such as metformin, sitagliptin, pioglitazone, and insulin glargine [23-25].

In addition to the parameters measured in the primary studies, measures that allow simplified comparisons of the ability of two different therapies to assist patients in reaching a treatment goal are of value. The number needed to treat (NNT) is one such measure that reflects the number of patients a clinician needs to treat with a particular therapy instead of another therapy (or compared to a control) to allow one additional patient to achieve a treatment goal. The NNT is calculated by comparing the relative percentages of patients reaching a treatment goal in each therapeutic group, also called the absolute benefit increase (ABI) [26-28].

The purpose of this analysis was to compare exenatide QW with several commonly prescribed therapies from other drug classes to assess the ABI and the associated NNT for achieving a clinically beneficial endpoint, defined as achieving a single or composite of ADA-recommended goals.

## Methods

Data from the intent-to treat (ITT) population of 3 randomized, controlled, 26-week, Phase 3 studies (DURATION-2, -3, and -4) were retrospectively analyzed separately [23-25]. The ITT population was defined as patients receiving at least one dose of the randomized study medication. Eligible patients in the trials had T2DM and were at least 18 years of age with a baseline HbA1c of 7.1 to 11.0%, a body mass index of 25-45 kg/m^2^, and a history of stable body weight prior to the screening visit. Each trial was performed in accordance with the ethics principles stated in the Declaration of Helsinki [29]. An ethics review board reviewed each study protocol before trial initiation and patients provided written consent before any procedure was performed. The studies are registered with ClinicalTrials.gov, clinical trial numbers NCT00637273, NCT00641056, and NCT00676338.

In all trials, standard doses of medications were administered according to local label approval. Patients in the double-blind, DURATION-2 study received a stable dose of metformin as background therapy and were randomized to receive 2 mg of exenatide QW, 100 mg/day oral sitagliptin, or 45 mg/day oral pioglitazone for 26 weeks. In the open-label, DURATION-3 study, patients using metformin with or without sulfonylurea as a background therapy were randomized to receive either 2 mg of exenatide QW or once daily insulin glargine, titrated to fasting blood glucose concentrations of 4.0 to 5.5 mmol/L, for 26 weeks. It was recommended that patients using sulfonylurea reduce their dose if confirmed hypoglycemia occurred. In the double-blind DURATION-4 study, drug-naïve patients were randomized to 2 mg of exenatide QW, 2000 mg/day metformin, 100 mg/day sitagliptin, or 45 mg/day pioglitazone for 26 weeks. Metformin and pioglitazone dosages were increased in weekly increments up to target doses; metformin could be increased to 2500 mg/day depending on glycemic control. Patient descriptions, randomization, and procedures were described in detail previously [23-25].

The attainment of target goals was used as surrogate endpoints for beneficial outcomes (Goal_x_) and included three single glycemic goals and four composite goals (Table 1). The ABI and NNT for the single components of the composite goals such as weight loss (or no weight gain), systolic blood pressure (SBP), low-density lipoprotein (LDL) cholesterol, and lack of hypoglycemia (hypoglycemia defined as documented blood glucose <3 mmol/L [54 mg/dL]) were also calculated. To quantitate the ABI for each selected goal, the percentage of ITT patients in either the exenatide QW or a comparator arm in each study who were not at the indicated goal at baseline but who reached the goal at endpoint (26-weeks) was calculated. The ABI was computed as:

$$\mathrm{ABI}=\text{\%}\;\mathrm{exenatide}\;\mathrm{QW}\;\mathrm{patients}\;\mathrm{reaching}\;\mathrm{Goa}{\text{l}}_{\text{x}}-\text{\%}\;\mathrm{comparator}\;\mathrm{patients}\;\mathrm{reaching}\;\mathrm{Goal}$$

Individual patient data was used in the analysis. The last post-baseline data were used to determine achievement of specific goals. Subjects with no post-baseline measurement were considered to have not reached the goal at endpoint.

The NNT indicates the number of patients that would need to be treated with exenatide QW vs a comparator therapy in order to have one additional patient reach a particular target goal. The NNT was calculated as 1/ABI and rounded to next highest integer. Confidence intervals of the NNT were calculated by taking the reciprocals of the values defining the 95% confidence interval (CI) for the ABI and rounding to the next highest integer [27]. The sign and magnitude of the NNT were used to determine the extent of the benefit (or lack of benefit) of treatment with exenatide QW. A positive NNT indicated an overall benefit in achieving a goal with exenatide QW use and a negative NNT number indicated the benefit of the comparator. In addition, the smaller the absolute NNT value, the larger the difference between the two treatment groups [30].

## Results

### Patient demographics

Across each individual study, the baseline characteristics for each therapeutic arm were generally similar (Table 2). However, all three DURATION studies had a slightly different population of patients. All patients in DURATION-2 were treated with concomitant metformin therapy whereas patients in DURATION-3 were treated with metformin with or without a sulfonylurea; patients in DURATION-4 were drug-naïve and suboptimally controlled with diet and exercise. The duration of diabetes was shorter in DURATION-4 compared to the other two studies. Additionally, the ethnic backgrounds were slightly different among the studies. There were more Black and Hispanic patients and fewer White patients in DURATION-2 than in DURATION-3 or -4, and there were fewer Asian patients in DURATION-3 than in the other two studies.

**Table 2:** Baseline demographics

	DURATION-4*				**DURATION-2†**			**DURATION-3‡**	
	**ExQW**	**Met**	**Sita**	**Pio**	**EQW**	**Sita**	**Pio**	**ExQW**	**IG**
**N**	248	246	163	163	160	166	165	233	223
**Sex (Male) (%)**	56	63	58	60	56	52	48	52	55
**Race (%)**									
**American Indian or Alaska Native**	<1	1	1	0	0	2	0	0	0
**Asian**	22	21	20	21	23	25	24	6	6
**Black or African American**	3	5	2	3	12	12	8	1	>1
**Hispanic**	7	9	8	9	31	30	27	12	9
**Other**	0	<1	0	0	1	1	2	0	0
**White**	68	65	69	68	33	30	39	82	85
**Age (y)**	54 ± 11	54 ± 11	52 ± 11	55 ± 11	52 ± 10	52 ± 11	53 ± 10	58 ± 10	58 ± 9
**Duration of diabetes (y)**	3 ± 3	3 ± 4	3 ± 4	3 ± 4	6 ± 5	5 ± 5	6 ± 6	8 ± 6	8 ± 6
**Baseline weight (kg)**	87 ± 19	86 ± 20	89 ± 19	86 ± 18	89 ± 20	87 ± 20	88 ± 21	91 ± 19	91 ± 16
**Baseline BMI (kg/m**^**2**^**)**	31 ± 5	31 ± 5	32 ± 5	31 ± 5	32 ± 5	32 ± 5	33 ± 6	32 ± 5	32 ± 5
**Baseline HbA1c (%)**	8.5 ± 1.2	8.6 ± 1.2	8.5 ± 1.3	8.5 ± 1.2	8.6 ± 1.2	8.5 ± 1.2	8.5 ± 1.1	8.3 ± 1.1	8.3 ± 1.0
**Baseline FBG (mmol/L)**	9.9 ± 2.9	10.0 ± 3.4	9.7 ± 2.6	9.8 ± 3.0	9.2 ± 2.9	9.1 ± 2.5	9.1 ± 2.4	9.9 ± 2.5	9.7 ± 2.7
**Baseline SBP (mm Hg)**	129 ± 12	129 ± 15	130 ± 13	131 ± 15	126 ± 14	126 ± 14	127 ± 14	135 ± 17	133 ± 16
**Baseline LDL (mmol/dL)**	3.1 ± 1.1	2.9 ± 0.9	3.0 ± 0.9	3.1± 1.0	2.6 ± 0.8	2.8 ± 0.9	2.9 ± 1.0	2.7 ± 0.9	2.7 ± 0.9
**Background therapy (n)**									
**Metformin**					160	166	165	164	157
**Diet and Exercise**	248	246	163	163					
**Metformin + Sulfonylurea**								69	66

*†‡**D**iabetes Therapy **U**tilization: **R**esearching Changes in **A**1C, Weight and Other Factors **T**hrough **I**ntervention with Exenatide **O**nce-**W**eekly-4, -2, -3 [23-25].

Values are shown as mean ± SD.

BMI , body mass index; DBP, diastolic blood pressure; ExQW, exenatide QW; HDL, high density lipoprotein cholesterol; IG, insulin glargine; Met, metformin; Pio, pioglitazone; Sita, sitagliptin.

### Exenatide QW vs metformin

Metformin is generally considered to be the first line therapy for patients with type 2 diabetes. The results of the DURATION -4 study showed that exenatide QW was noninferior to metformin and that metformin and exenatide QW provided similar improvements in glycemic control (-1.5% vs -1.5%) in treatment-naïve patients, with the added benefits of weight reduction and minimal risk of hypoglycemia [25]. The results of the present analysis reaffirm the original results; for most goals, both therapies had similar effects.

**Figure 1:**
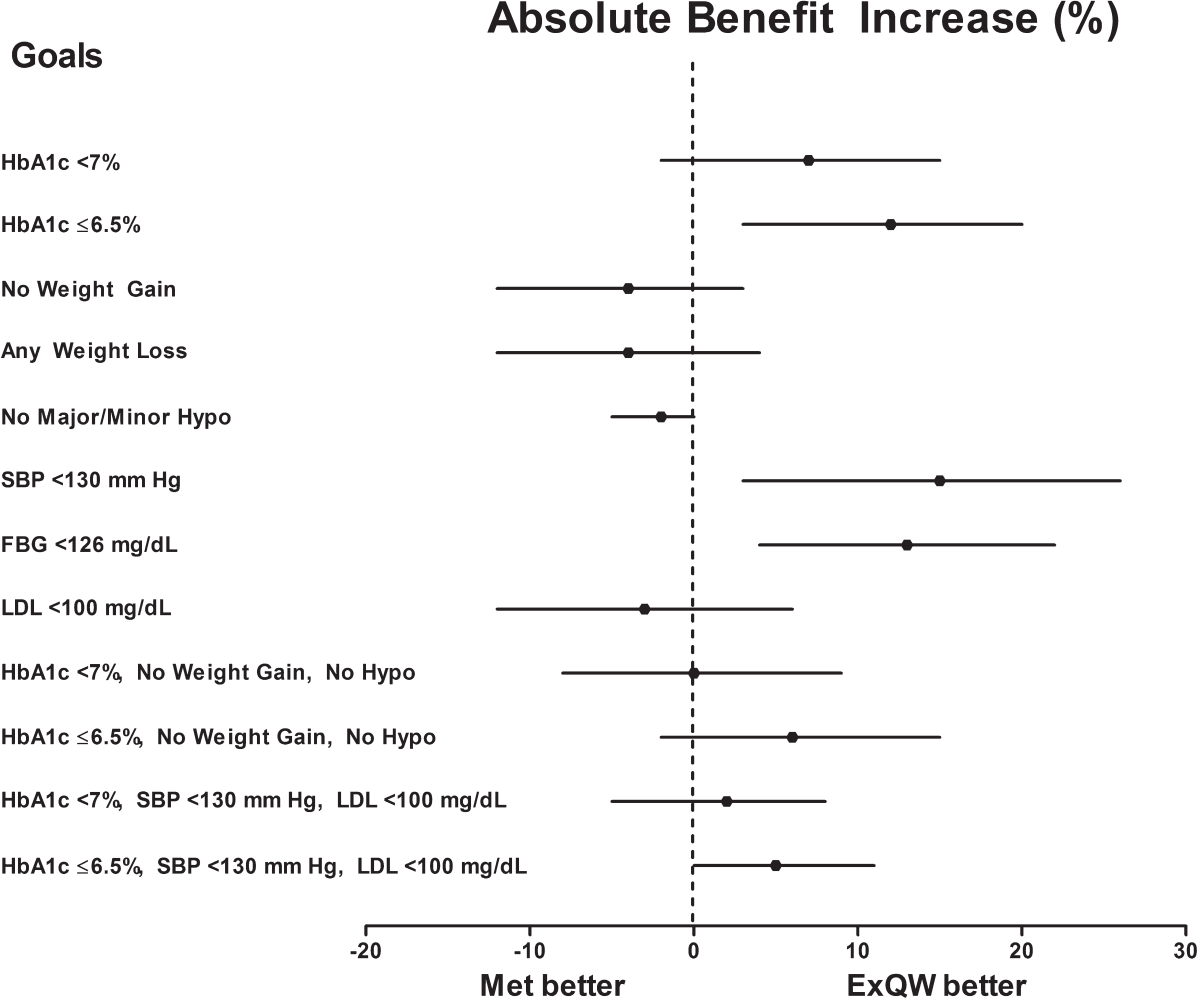
**Absolute Benefit Increase (ABI) of Exenatide QW (ExQW) vs Metformin.** Forest plot depicts the ABI (%) ± 95% CI of ExQW vs metformin. ABI = 0% indicates no benefit; ABI <0% indicates metformin provides a benefit vs ExQW.

The ABI [95% CI] significantly favored exenatide QW over metformin for the glycemic goals of HbA1c ≤6.5% and fasting blood glucose (FBG) <6.99 mmol/L (126 mg/dL) (12% [3%, 20%] and 12.9% [4%, 22%], respectively), with associated NNTs of 9 and 8, respectively (Table 3 and Figure 1). None of the composite goals showed a significant difference between the two therapies.

**Table 3:** Absolute benefit increase and number needed to treat by comparator and study

**Single Goals**									
	**DURATION-4**			**DURATION-2**			**DURATION-3**		
**Therapy**	**% Reaching Goal (N)**	**ABI** **(95% CI)**	**NNT** **(95% CI)**	**% Reaching Goal (N)**	**ABI** **(95% CI)**	**NNT** **(95% CI)**	**% Reaching Goal (N)**	**ABI** **(95% CI)**	**NNT** **(95% CI)**
**HbA1c <7.0%**									
**ExQW**	61.6%			58.6%			59.0%		
	(237)			(157)			(222)		
**Met**	54.8%	6.8%	15						
	(239)	(-2%, 15%)	(7, ∞, -49)						
**Sita**	41.4%	20.2%	5	27.7%	30.9%	4			
	(152)	(10%, 30%)	(4, 10)	(155)	(20%, 41%)	(3, 5)			
**Pio**	58.5%	3.0%	34	43.4%	15.2%	7			
	(157)	(-7%, 13%)	(8, ∞, -15)	(159)	(4%, 26%)	(4, 24)			
**IG**							46.5%	12.5%	8
							(215)	(3%, 22%)	(5, 32)
**HbA1c ≤6.5%**									
**ExQW**	48.2%			38.8%			42.2%		
	(247)			(160)			(232)		
**Met**	36.2%	12.0%	9						
	(243)	(3%, 20%)	(5, 31)						
**Sita**	24.5%	23.7%	5	14.6%	24.2%	5			
	(163)	(14%, 32%)	(4, 7)	(164)	(15%, 33%)	(3, 7)			
**Pio**	40.9%	7.3%	14	26.1%	12.7	8			
	(159)	(-3%, 17%)	(6, ∞, -38)	(165)	(3%, 23%)	(5, 40)			
**IG**							28.1%	14.1%	8
							(221)	(5%, 23%)	(5, 19)
**No Weight Gain**									
**ExQW**	72.6%			78.1%			84.1%		
	(248)			(160)			(233)		
**Met**	76.8%	-4.2%	-24						
	(246)	(-12%, 3%)	(29, ∞, -9)						
**Sita**	65.0%	7.6%	14	59.6%	18.5%	6			
	(163)	(-1%, 17%)	(6, ∞, -69)	(166)	(8%, 28%)	(4, 12)			
**Pio**	36.8%	35.8%	3	24.2%	53.9%	2			
	(163)	(26%, 45%)	(3, 4)	(165)	(44%, 62%)	(2, 3)			
**IG**							33.2%	50.9%	2
							(223)	(43%, 58%)	(2, 3)
**Any Weight Loss**									
**ExQW**	68.5%			76.9%			79.0%		
	(248)			(160)			(233)		
**Met**	72.8%	-4.3%	-24						
	(246)	(-12%, 4%)	(27, ∞, -9)						
**Sita**	55.9%	12.7%	8	59.0%	17.9%	6			
	(163)	(3%, 22%)	(5, 32)	(166)	(8%, 27%)	(4, 13)			
**Pio**	33.1%	35.4%	3	21.2%	55.7%	2			
	(163)	(26%, 44%)	(3, 4)	(165)	(46%, 64%)	(2, 3)			
**IG**							30.5%	48.5%	3
							(223)	(40%, 56%)	(2, 3)
**No Major/Minor Hypoglycemia**									
**ExQW**	98.0%			98.8%			88.4%		
	(248)			(160)			(233)		
**Met**	100.0%	-2.0%	-50						
	(246)	(-5%, 0%)	(∞, -22)						
**Sita**	100.0%	-2.0%	-50	97.0%	1.8%	56			
	(163)	(-5%, 1%)	(175, ∞, -22)	(166)	(-2%, 6%)	(17, ∞, -56)			
**Pio**	100.0%	-2.0%	-50	98.8%	0	N/A			
	(163)	(-5%, 1%)	(175, ∞, -22)	(165)	(-3%, 3%)				
**IG**							69.5%	18.9%	6
							(223)	(12%, 26%)	(4, 9)
**SBP<130 mm Hg**									
**ExQW**	39.7%			56.9%			25.3%		
	(126)			(58)			(154)		
**Met**	25.0%	14.7%	7						
	(124)	(3%, 26%)	(4, 32)						
**Sita**	31.2%	8.5%	12	34.8%	22.1%	5			
	(93)	(-4%, 21%)	(5, ∞, -23)	(66)	(5%, 38%)	(3, 22)			
**Pio**	32.2%	7.5%	14	39.1%	17.8%	6			
	(87)	(-6%, 20%)	(5, ∞, -18)	(69)	(0%, 34%)	(3, 244)			
**IG**							21.3%	4.0%	25
							(136)	(-6%, 14%)	(8, ∞, -17)
**FBG <6.99 mmol/L (<126 mg/dL)**									
**ExQW**	47.0%			54.9%			38.6%		
	(219)			(122)			(215)		
**Met**	34.1%	12.9%	8						
	(217)	(4%, 22%)	(5, 27)						
**Sita**	20.8%	26.2%	4	27.4%	27.5%	4			
	(144)	(16%, 35%)	(3, 7)	(135)	(16%, 38%)	(3, 7)			
**Pio**	48.3%	-1.3%	-77	44.5%	10.4%	10			
	(145)	(-12%, 9%)	(11, ∞, -9)	(137)	(-2%, 22%)	(5, ∞, -57)			
**IG**							51.5%	-12.9%	-8
							(200)	(-22%, -3%)	(-5, -30)
**LDL <2.59 mmol/L (100 mg/dL)**									
**ExQW**	20.2%			29.8%			32.3%		
	(168)			(84)			(130)		
**Met**	23.1%	-2.9%	-35						
	(160)	(-12%, 6%)	(17, ∞, -9)						
**Sita**	13.8%	6.4%	16	21.6%	8.2%	13			
	(109)	(-3%, 15%)	(7, ∞, -33)	(97)	(-4%, 21%)	(5, ∞, -22)			
**Pio**	13.0%	7.2%	14	19.4%	10.4%	10			
	(115)	(-2%, 16%)	(7, ∞, -52)	(103)	(-2%, 23%)	(5, ∞, -53)			
**IG**							15.3%	17.0%	6
							(124)	(7%, 27%)	(4, 15)
**Composite Goals**									
**HbA1c <7.0%, No Weight Gain, No Hypoglycemia**									
**ExQW**	48.4%			48.1%			46.8%		
	(248)			(160)			(233)		
**Met**	48.0%	0.4%	250						
	(246)	(-8%, 9%)	(11, ∞, -12)						
**Sita**	34.4%	14.0%	8	22.3	25.8%	4			
	(163)	(4%, 23%)	(5, 24)	(166)	(16%, 35%)	(3, 7)			
**Pio**	21.5%	26.9%	4	9.7%	38.4%	3			
	(163)	(18%, 35%)	(3, 6)	(165)	(29%, 47%)	(3, 4)			
**IG**							13.0%	33.8%	3
							(223)	(26%, 41%)	(3, 4)
**HbA1c ≤6.5%, No Weight Gain, No Hypoglycemia**									
**ExQW**	39.1%			32.5%			30.5%		
	(248)			(160)			(233)		
**Met**	32.9%	6.2%	17						
	(246)	(-2%, 15%)	(7, ∞, -44)						
**Sita**	19.6%	19.5%	6	12.0%	20.5%	5			
	(163)	(11%, 28%)	(4, 10)	(166)	(12%, 29%)	(4, 9)			
**Pio**	17.2%	21.9%	5	5.5%	27.0%	4			
	(163)	(13%, 30%)	(4, 8)	(165)	(19%, 35%)	(3, 6)			
**IG**							9.4%	21.1%	5
							(223)	(14%, 28%)	(4, 8)
**HbA1c<7%, SBP <130 mm Hg, LDL <2.59 mmol/L**									
**ExQW**	15.0%			22.6%			17.7%		
	(246)			(159)			(231)		
**Met**	13.4%	1.6%	63						
	(246)	(-5%, 8%)	(13, ∞, -22)						
**Sita**	5.0%	10.0%	10	11.6%	11.0%	10			
	(160)	(4%, 16%)	(7, 25)	(164)	(3%, 19%)	(6, 36)			
**Pio**	8.7%	6.3%	16	14.6%	8.0%	13			
	(161)	(0%, 12%)	(9, ∞, -285)	(164)	(-1%, 16%)	(7, ∞, -197)			
**IG**							10.3%	7.4%	14
							(223)	(1%, 14%)	(8, 101)
**HbA1c ≤6.5%, SBP<130 mm Hg, LDL <2.59 mmol/L**									
**ExQW**	12.9%			16.9%			12.9%		
	(248)			(160)			(232)		
**Met**	7.7%	5.2%	20						
	(246)	(0%, 11%)	(10, ∞, -490)						
**Sita**	4.3%	8.6%	12	6.1%	10.8%	10			
	(163)	(3%, 14%)	(8, 34)	(165)	(4%, 18%)	(6, 26)			
**Pio**	6.2%	6.7%	15	9.1%	7.8%	13			
	(161)	(1%, 12%)	(9, 152)	(165)	(0%, 15%)	(7, 220 )			
**IG**							5.8%	7.1%	15
							(223)	(2%, 13%)	(8, 58)

ABI, Absolute Benefit Increase; NNT, number needed to treat;

NNT compares ExQW with indicated comparator; an infinity limit (∞) is shown for the 95% CI of the NNT if the 95% CI of the ABI contains zero;

N refers to subjects not at goal at baseline.

### Exenatide QW vs sitagliptin

In two of the Phase 3 studies, DURATION-2 and DURATION-4, the safety and efficacy of exenatide QW was compared to sitagliptin in two different populations of patients (metformin co-treatment or treatment-naïve, respectively). The primary outcome of the two trials showed that exenatide QW was superior to sitagliptin in reducing HbA1c, with LS mean reductions in HbA1c of -1.5% (exenatide QW) vs -0.9% (sitagliptin) in DURATION-2, and -1.5% (exenatide QW) vs -1.2% (sitagliptin) in DURATION-4 [23,25].

**Figure 2:**
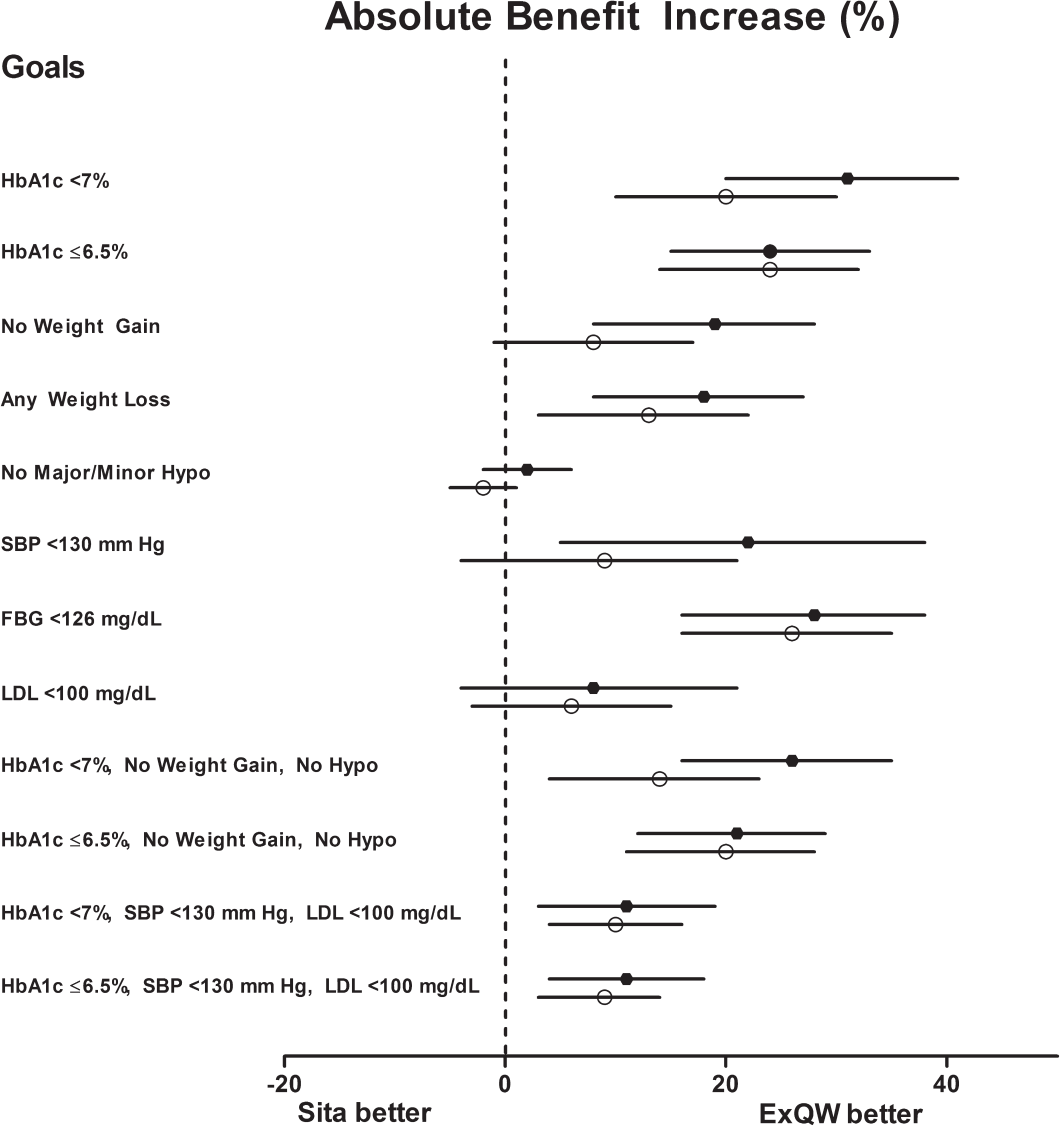
**Absolute Benefit Increase (ABI) of Exenatide QW (ExQW) vs Sitagliptin.** Forest plot depicts the ABI (%) ± 95% CI of ExQW vs sitagliptin. Data comparing ExQW to sitagliptin originate from DURATION-2 (filled circles) and DURATION-4 (open circles) studies. ABI = 0% indicates no benefit; ABI <0% indicates sitagliptin provides a benefit vs ExQW.

Consistent with the findings of a greater reduction in HbA1c with exenatide QW compared to sitagliptin, a larger percentage of patients treated with exenatide QW compared to sitagliptin attained the HbA1c goals of <7% or ≤6.5% (Table 3). The largest benefit for the use of exenatide QW vs sitagliptin was observed in attaining the goal of HbA1c <7% (in DURATION-2) where the ABI (95% CI) was 30.9% (20%, 41%). The ABI favored exenatide QW over sitagliptin for each HbA1c and FBG single goal and for all of the composite goals in both studies (Figure 2). The associated NNTs for each of the goals were generally similar in both DURATION-2 and -4 studies (Table 3). Five patients would need to be treated with exenatide QW rather than sitagliptin for 26 weeks to have one additional patient reach an HbA1c goal of ≤6.5%. Similarly, the NNT for one additional patient to attain the composite goal of HbA1c ≤6.5% without weight gain or hypoglycemia was 5 to 6. In both studies comparing exenatide QW and sitagliptin, the NNT was 10 for the composite goal combining HbA1c <7%, SBP <130 mm Hg, and LDL <2.59 mmol/L.

### Exenatide QW vs pioglitazone

The DURATION-2 and DURATION-4 studies also compared exenatide QW with pioglitazone. The reduction in HbA1c with exenatide QW treatment was consistent in both trials (-1.5%), while the reduction in HbA1c with pioglitazone treatment was -1.2% in DURATION-2 (on a background of metformin-treated patients) and -1.6% in DURATION-4 (in a drug-naïve patient population); resulting in exenatide QW superiority over pioglitazone in DURATION-2 and lack of noninferiority in DURATION-4.

**Figure 3:**
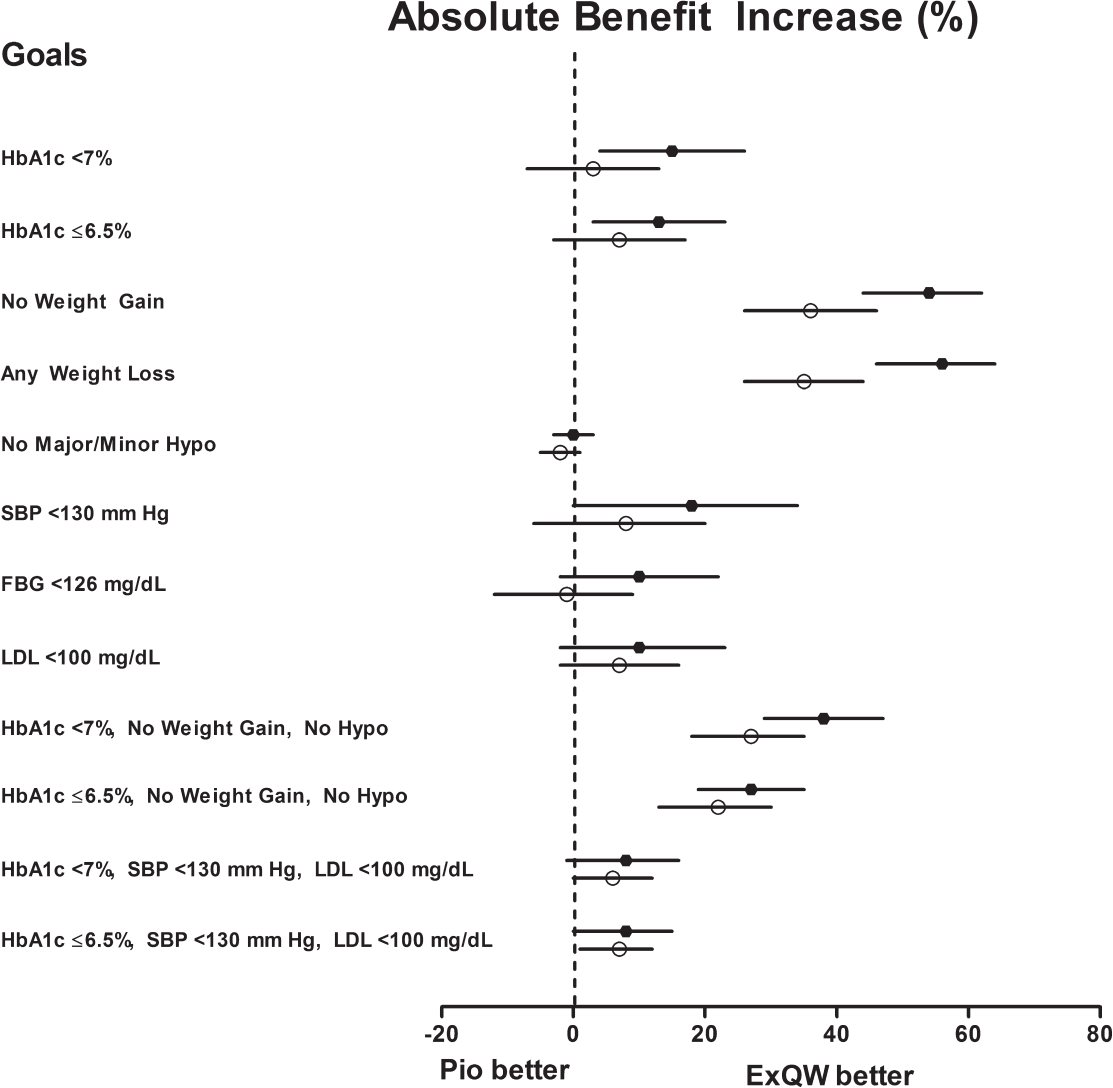
**Absolute Benefit Increase (ABI) of Exenatide QW (ExQW) vs Pioglitazone.** Forest plot depicts the ABI (%) ± 95% CI of ExQW vs pioglitazone. Data comparing ExQW to pioglitazone originate from DURATION-2 (filled circles) and DURATION-4 (open circles) studies. ABI = 0% indicates no benefit; ABI <0% indicates pioglitazone provides a benefit vs ExQW.

The ABI for both HbA1c single goals favored exenatide QW over pioglitazone in both studies; however, the results were not significant in the DURATION-4 study (Table 3 and Figure 3). The ABI for FBG in the DURATION-4 study slightly favored pioglitazone (-1.3% [-12%, 9%]) but the result was also not significant (Figure 3). Although the primary result of the DURATION-4 study failed to demonstrate noninferiority of exenatide QW vs pioglitazone with regard to HbA1c reduction, the ABI for the composite goals of HbA1c at target (<7% or ≤6.5%) with no weight gain or hypoglycemia, significantly favored exenatide QW (HbA1c <7% composite goal: 26.9% [18%, 35%]; HbA1c ≤6.5% composite goal: 21.9% [13%, 30%]) with an associated NNT of 4 and 5, respectively. The NNT for these same composite goals was 3 and 4, respectively in the DURATION-2 study.

### Exenatide QW vs insulin glargine

**Figure 4:**
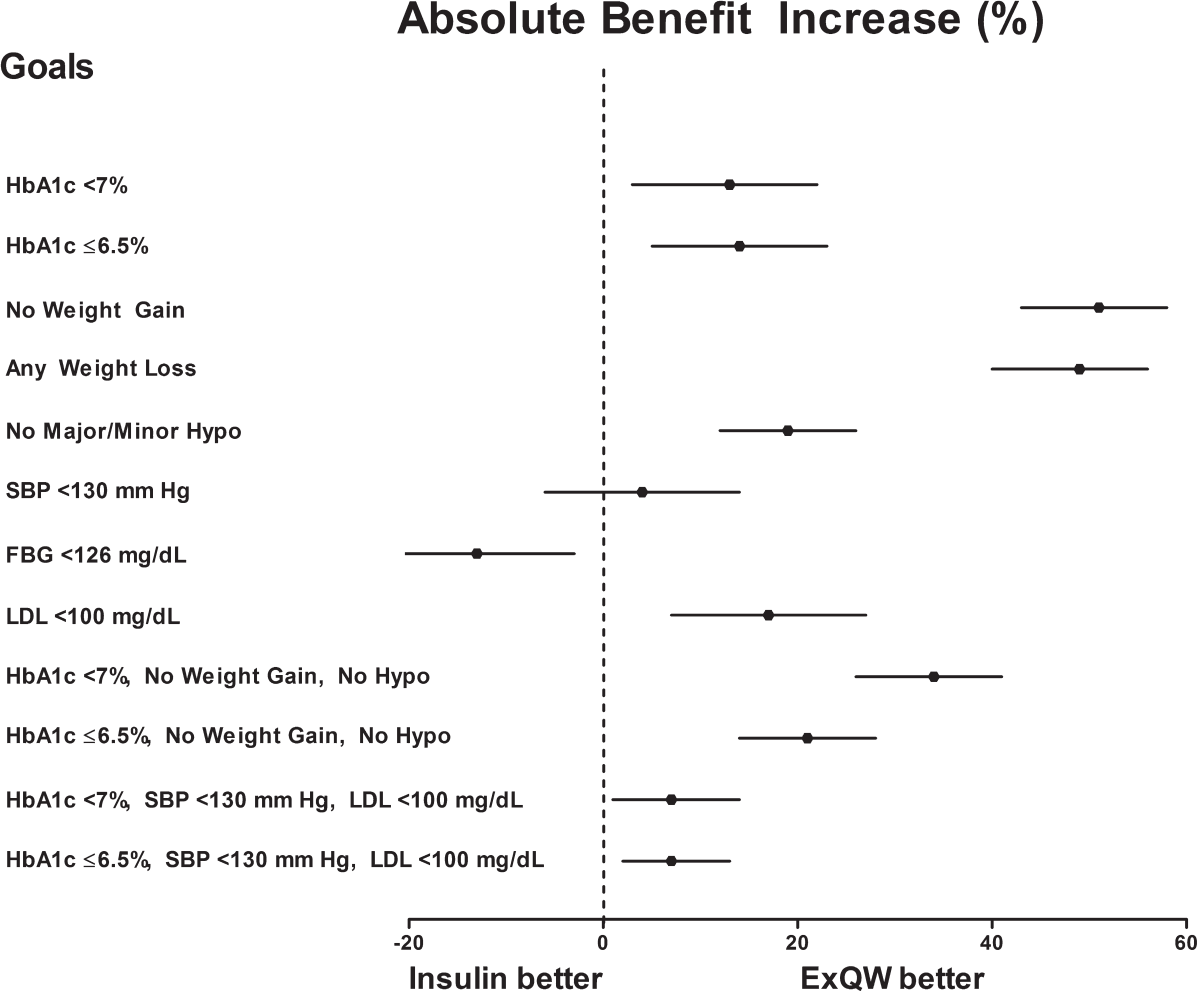
**Absolute Benefit Increase (ABI) of Exenatide QW (ExQW) vs Insulin Glargine.** Forest plot depicts the ABI (%) ± 95% CI of ExQW vs insulin glargine. ABI = 0% indicates no benefit; ABI <0% indicates insulin glargine provides a benefit vs ExQW.

The efficacy and safety of exenatide QW vs insulin glargine was compared in the DURATION-3 study resulting in a reduction in HbA1c of -1.5% for exenatide QW and -1.3% for insulin glargine [24]. In the current analysis, a larger percentage of patients in the exenatide QW group reached the recommended HbA1c goals of <7.0% and ≤6.5% compared to patients in the insulin group (Table 3). The resulting ABI was 12.5% (3%, 22%) and 14.1% (5%, 23%), respectively, with an NNT of 8 for both goals. A larger percentage of patients in the insulin glargine group attained the FBG goal, resulting in an ABI of -12.9% (-22%, -3%) and an associated NNT of -8 (Table 3, Figure 4). Exenatide QW was significantly favored over insulin glargine for attaining all four composite goals (Figure 4). Of all of the composite goals, the largest benefit of exenatide QW use was observed for the goal of HbA1c <7% without weight gain or hypoglycemia with an ABI of 33.8% (26%, 41%) and an associated NNT of 3 (Figure 4).

### Adverse events

The most frequent adverse events occurring in patients treated with exenatide QW, metformin, sitagliptin, pioglitazone, or insulin glargine in each of the three studies is listed in Table 4. Across the studies, the most common adverse events for any of the treatments in all studies were nausea, diarrhea, injection site reactions, nasopharyngitis, and headache. The incidence of adverse events leading to withdrawal was 7%, 3%, and 4% (exenatide QW, sitagliptin, and pioglitazone, respectively) in DURATION-2, 5% and 1% (exenatide QW and insulin glargine, respectively) in DURATION-3, and 2%, 2%, 1% and 3% (exenatide QW, metformin, sitagliptin, and pioglitazone, respectively) in DURATION-4.

**Table 4:** Incidence of adverse events >5% in any treatment group in any study

	**DURATION**-4*				**DURATION-2†**			**DURATION-3‡**	
	**ExQW**	**Met**	**Sita**	**Pio**	**ExQW**	**Sita**	**Pio**	**ExQW**	**IG**
	**(N = 248)**	**(N = 246)**	**(N = 163)**	**(N = 163)**	**(N = 160)**	**(N = 166)**	**(N = 165)**	**(N = 233)**	**(N = 223)**
**Arthralgia (%)**	5	1	2	3	3	5	2	4	3
**Back pain (%)**	2	6	3	4	4	4	3	4	2
**Constipation (%)**	9	3	3	2	6	2	1	3	2
**Diarrhea (%)**	11	13	6	4	18	10	7	9	4
**Dyspepsia (%)**	7	3	2	5	4	4	2	3	1
**Fatigue (%)**	1	0	1	1	6	0	3	3	<1
**Headache (%)**	8	12	9	8	9	9	4	10	7
**Hypertension (%)**	1	1	2	7	3	3	1	2	3
**Injection site nodule (%)**	11	10	7	4	3	1	1	6	0
**Injection site pruritus (%)**	4	2	1	1	5	5	1	1	<1
**Nasopharyngitis (%)**	8	5	10	9	4	2	3	13	18
**Nausea (%)**	11	7	4	4	24	10	5	13	1
**Peripheral edema (%)**	0	<1	1	7	1	3	8	0	0
**Sinusitis (%)**	<1	<1	0	2	3	1	7	1	2
**Urinary tract infection (%)**	1	2	1	1	6	5	4	1	<1
**Upper-respiratory tract infection (%)**	3	3	1	2	4	9	10	2	1
**Vomiting (%)**	5	3	2	3	11	2	3	4	1

ND, not determined.

Data are% of intent-to-treat population.

*†‡**D**iabetes Therapy **U**tilization: **R**esearching Changes in **A**1C, Weight and Other Factors **T**hrough **I**ntervention with Exenatide **O**nce-**W**eekly-4, -2, -3 [23-25].

## Discussion

The selection of an optimal agent for treatment of T2DM can pose a challenge given that many therapeutic options are effective in reducing HbA1c, an indicator of blood glucose control. However, not all therapies reduce HbA1c sufficiently to achieve therapeutic goals. The recent Standards in Medical Care in Diabetes from the ADA has outlined a set of target goals believed to favorably affect the health outcomes of patients with T2DM [11]. The guidelines also suggest that individual preferences and comorbidities should be taken into consideration when choosing a therapy. Factors such as weight loss (or at least no weight gain) and the avoidance of hypoglycemia are important considerations. Indeed, in a patient survey conducted in patients with T2DM, more than half of the patients indicated that they would be willing to take an injectable once-weekly medication if the medication promoted weight loss or helped avoid weight gain [31]. Hypertension and dyslipidemia are other major modifiable risk factors for CVD commonly associated with T2DM. As such, they contribute to the already increased risk of CVD. Beyond glycemic control, the improvement of such CV risk factors represents an added advantage to therapies providing such benefits.

### Achievement of glycemic goals

This analysis compared the ability of exenatide QW vs metformin, sitagliptin, pioglitazone, or insulin glargine to assist patients in achieving ADA-recommended target goals. NNTs were calculated as a metric to assess how many patients would need to be treated with one therapy rather than another to allow one additional patient to reach a target. Compared to the other therapies examined, exenatide QW assisted a larger proportion of patients who were not already at HbA1c ≤6.5% or <7% at baseline in reaching the target goal after 26 weeks of treatment. The ABI for the goal of HbA1c ≤6.5% significantly favored exenatide QW over metformin, sitagliptin, pioglitazone, or insulin glargine in all studies except for DURATION-4 where the ABI for exenatide QW vs pioglitazone favored exenatide QW, but did not reach statistical significance. Contrary to the HbA1c goals, insulin was significantly favored over exenatide QW for the FBG goal. One reason for the discrepancy between the two glycemic endpoints is that the insulin was titrated according to a FBG target. Thus, the dosage of insulin glargine was raised until the FBG target was met, thereby contributing to bias. In addition, FBG is a short-term measure of blood glucose levels while HbA1c indirectly measures an average glucose concentration over a prolonged period of time. As insulin glargine is a titrated, long-acting basal insulin, it may function well to reduce fasting glucose levels but permit higher postprandial excursions leading to a larger average glucose concentration compared to the glucose-dependent activity of exenatide QW [32,33].

The results of this analysis provide information to assist in personalization of therapy. The NNT for the HbA1c ≤6.5% goal was 8 when exenatide QW was compared to insulin glargine. However, for the alternate glycemic goal, FBG <6.99 mmol/L, the NNT was -8 for exenatide QW vs insulin glargine (favoring insulin glargine). This means that, in patients for whom FBG may be the more important clinical concern, treatment of 8 patients with insulin glargine rather than exenatide QW would allow one additional patient to reach a FBG goal. However, in patients wishing to achieve the HbA1c target (≤6.5%), 8 patients would need to be treated with exenatide QW rather than insulin to allow one additional patient to achieve the goal.

### Control of cardiovascular risk factors

Although the main focus of treatment is a reduction in hyperglycemia, improved glycemic control is associated with weight gain and the risk of hypoglycemia for some medications, and these CV risk factors also reduce patients’ quality of life. Unlike medications that promote weight gain or increase the risk of hypoglycemia, GLP-1 receptor agonists are generally associated with weight loss and minimal risk of hypoglycemia. In the current analysis, examination of the weight and hypoglycemia effects of exenatide QW was assessed as a composite goal of HbA1c <7% with no weight gain and no hypoglycemia. Exenatide QW provided a significant ABI for achieving this composite goal compared to sitagliptin, pioglitazone, and insulin glargine, with NNTs as low as 3 (vs pioglitazone and insulin glargine). Across the three studies, exenatide QW assisted between 47 and 48% of patients who were not already at this composite goal in achieving the goal after 26 weeks. The comparators assisted between 10% (pioglitazone) and 48% (metformin) of patients in achieving this goal. These data are consistent with an analysis pooling 804 patients treated with exenatide QW during the DURATION trials, where 50% of patients treated with exenatide QW achieved the goal of HbA1c <7% with no weight gain and no hypoglycemia after 24-30 weeks [34]. A recent meta-analysis study similarly showed that 1.8 mg liraglutide once daily assisted 40% of patients in achieving the goal of HbA1c <7% with no weight gain and no hypoglycemia [35]. The meta-analysis additionally showed that the twice-daily formulation of exenatide assisted 25% of patients in achieving the composite goal, which was a higher percentage than sulfonylureas (8%), thiazolidinediones (6%), insulin glargine (15%), sitagliptin (11%), and placebo (8%) [35].

The magnitudes of the NNT values obtained for some goals reflect key differences between therapies. To put NNT values into better perspective, an NNT of 1 would mean that every patient treated would reach the specified goal. The significant NNTs for the composite goal of HbA1c <7% with no weight gain and no hypoglycemia ranged from 3 when exenatide QW was compared to insulin glargine and pioglitazone (in DURATION-2) to 8 when exenatide QW was compared to sitagliptin in DURATION-4. However, an NNT of 250 was observed for exenatide QW compared to metformin suggesting no greater benefit of either treatment in achieving this composite goal. As commonly used, an NNT of 20 or less is generally sufficient to justify the choice of a clinical intervention in the absence of a more significant clinical harm [30,36].

A second composite goal, based on findings from the Steno-2 study, highlighted the importance of HbA1c, blood pressure, and lipids in the reduction of CV risk in patients with T2DM [4]. The Steno-2 study identified the benefits of a multifactorial intervention strategy by demonstrating an approximately 50% reduction in risk of CV and microvascular events by targeting multiple risk factors with intensified treatment [4]. Despite the importance of such a multi-targeted therapeutic approach, the NHANES analysis reported that between 1999 and 2004, only 13% of surveyed diabetes patients achieved a composite goal of HbA1c <7.0%, blood pressure <130/80 mm Hg, and total cholesterol <5.17 mmol/L (<200 mg/dL) [37]. The current analysis used the individual ADA-suggested goals to define a composite goal of HbA1c <7%, SBP <130 mm Hg, and LDL <2.59 mmol/L (<100 mg/dL). The results showed that across the three studies, between 23% and 15% of patients who were not already at the goal at baseline reached the composite goal using exenatide QW, slightly more than observed in the NHANES report. Comparators assisted between 15% (pioglitazone, DURATION-2) and 5% (sitagliptin, DURATION-4) of patients in reaching the composite goal which is in line with NHANES estimates for this composite. The ABI for this goal significantly favored exenatide QW compared to sitagliptin or insulin glargine with NNTs of 10 and 14, respectively.

### Limitations and additional studies

Limitations of the current analysis include its retrospective, post hoc design and the small sample size. Common NNT analyses generally examine larger populations over a longer time period to observe a particular rare but clinically important outcome in a significant number of patients [28,38,39]. A study of the attainment of therapeutic goals and ABI calculations in a large, real-world population would be a useful follow-up to this study. Since exenatide QW was approved in 2011 in Europe and in 2012 in the United States, substantive real-world data are not yet available. Furthermore, the second composite goal (HbA1c <7%, SBP <130 mm Hg, and LDL <2.59 mmol/L) focused on a single lipid parameter, whereas other lipid parameters (eg, triglycerides, high-density lipoprotein cholesterol, lipoprotein subfractions) and endothelial function also influence CV risk and have been shown to be affected by treatment with exenatide QW, but evaluation of these variables were beyond the scope of the current investigation [40-42]. In addition, NNTs were calculated in the absence of a number needed to harm as the common adverse events associated with each of the therapies examined here were therapy-specific.

The current analysis comparing glucose-lowering therapies does not take into account differential cost or convenience factors; nor does it consider patient or physician perception of the different routes of administration. In addition, although the composite goals examined provide a clinically relevant combination of treatment targets, a single component of the composite could drive the result. For example, pioglitazone and insulin glargine are often associated with weight gain and therefore would not be favored in achieving composite goals containing a weight neutral component. Likewise, since insulin glargine is a glucose-independent, titrated medication, there is a greater risk for hypoglycemia which would lead to an unfavorable comparison with a glucose-dependent therapy (such as exenatide QW) in a composite goal including no hypoglycemia.

Although surrogate endpoints are not typically used in NNT assessments, the ability to achieve therapeutic goals is clinically important and relevant for short-term treatment decisions. Indeed, in a recent 1-year study with liraglutide, the NNT to achieve a loss of 10% of total body weight was calculated as 2 with liraglutide compared to 7 for placebo [43]. While the NNTs calculated in the current analysis reflect the attainment of a glycemic goal over a relatively short period of time (26 weeks), data has suggested that early intensive glucose control, even for a short period of time, might have a significant effect on CV events later in life, the so called legacy effect [44-46]. The ongoing EXSCEL (Exenatide Study of Cardiovascular Event Lowering, clinical trial number NCT01144338) trial will measure the time to the first confirmed CV event. In addition, the results of the trial are expected to further clarify the ability of exenatide QW to assist patients in reaching ADA-recommended goals and examine the presumption that reaching these goals correlates with a reduction of CV risk.

## Conclusions

This analysis compares the ability of exenatide QW vs commonly used glucose-lowering therapies representative of other drug classes (eg, biguanides, dipeptidyl peptidase-4 inhibitors, thiazolidinediones, or basal insulin) to assist patients in reaching important therapeutic goals. The results from clinical trials indicate that for the majority of ADA-recommended therapeutic goals, exenatide QW assists more patients in reaching the goal than treatment with sitagliptin, pioglitazone, or insulin glargine. The NNTs provided here indicate that fewer patients need to be treated with exenatide QW than with sitagliptin, pioglitazone or insulin glargine to attain the same composite goal of glycemic control without weight gain or hypoglycemia. The attainment of ADA-recommended goals promise to provide a clinical benefit in the reduction of CV risk and ongoing CV clinical trials are expected to provide answers as to whether reaching target goals are reliable predictors of reduced morbidity and mortality.

## Abbreviations

ABI: Absolute benefit increase; ADA: American Diabetes Association; ADVANCE: Action in Diabetes and Vascular Disease: Preterax and Diamicron MR Controlled Evaluation; ACCORD: Action to Control Cardiovascular Risk in Diabetes Trial; CI: Confidence interval; CV: Cardiovascular; CVD: Cardiovascular disease; DCCT: Diabetes Control and Complications Trial; DURATION: Diabetes Therapy Utilization: Researching Changes in A1C, Weight, and Other Factors Through Intervention with Exenatide Once Weekly; EXSCEL: Exenatide Study of Cardiovascular Event Lowering; FBG: Fasting blood glucose; GLP-1: Glucagon-like peptide-1; HbA1c: Glycated hemoglobin A1c; ITT: Intent-to-treat; LDL: Low density lipoprotein; NHANES: National Health and Nutrition Examination Survey; NNT: Number needed to treat; QW: Once weekly; SBP: Systolic blood pressure; T2DM: Type 2 diabetes mellitus; UKPDS: UK Prospective Diabetes Study.

## Competing interests

During the development of this manuscript, ARM, MBD, JH, JB, and MG were employees and stock holders of Amylin Pharmaceuticals, Inc. Amylin Pharmaceuticals, LLC is now a wholly owned subsidiary of Bristol-Myers Squibb.

## Authors’ contributions

ARM, MBD, JH, JB, and MG participated in the design of the analysis. JH performed the statistical analysis. All authors were involved in the interpretation of the analysis as well as drafting or critically revising the manuscript for important intellectual content. All authors read and approved the final manuscript.
